# Does implantation of an Amplatzer septal occluder (ASO) device change ventricular contraction pattern? An MR tagging study

**DOI:** 10.1186/1532-429X-11-S1-P209

**Published:** 2009-01-28

**Authors:** Heiko Stern, Sohrab Fratz

**Affiliations:** Deutsches Herzzentrum Munchen, München, Germany

**Keywords:** Right Ventricle, Atrial Septal Defect, Short Axis Slice, Atrial Septal Defect, Contraction Pattern

Amplatzer occluder devices are used throughout the world for occlusion of atrial septal defects (ASD) or persistent foramen ovale (PFO). This foreign body remains in place for life time and could change the contraction pattern of the heart. The contraction pattern should be analyzed by MR tagging. In order to differentiate motion changes by unloading the right ventricle (RV) from changes by the presence of the ASO itself both patients with ASD and PFO were included, presuming that PFO occluders did not change ventricular volumes.

## Patients

16 pts with ASD and 9 pts with PFO were investigated by MR tagging before and after defect closure by ASO. None of the patients had additional heart disease or heart block.

## Methods

MR tagging was performed for both RV and LV. Short axis cuts at three different levels of the ventricles were performed and an EPI-CSPAMM sequence (TE 7.5 ms, EPI Factor 11, Matrix 256 × 128, α = 30°) used. Data analysis based on HARP. Rotation of both ventricles was measured during systole and diastole and rotation was summed up and given in degrees of total ventricular rotation. Torsion was defined as difference in rotation between base and apex of the heart.

Circumferential strain (CS) and radial shortening (RS) of RV and LV were measured. Ventricular volumes were calculated using contiguous short axis slices of RV and LV, stroke volumes of both ventricles were measured by MR flow measurement in aorta and pulmonary artery.

## Results

Qp/Qs was reduced significantly by ASO implantation in the ASD group (p < 0,002), but remained unchanged in PFO patients. Accordingly RV enddiastolic volume was reduced (p < 0,05) and LV volumes increased significantly (p < 0,005) by occluding the ASD, but volumes remained unchanged after implanting PFO occluders.

Absolute changes in tagging parameters are listed in Table 1. Indicated are median values.

**Table 1:** Table 1

	ASD		PFO	
	RV	LV	RV	LV
Total rotation (°)	-0,8	-3,4 *	-1,0	-0,9
Torsion (°)	-0,4	0	0	-0,3
Circumferential Strain (%)	-2,2	+0,6	-1,9	-1,4
Radial shortening (%)	-2,8*	+0,6	-1,1	-1,7

* = p < 0.05

## Conclusion

There were significant changes in ventricular contraction pattern after closure of the ASD by an Amplatzer occluder. This is however due to load changes in both ventricles and not due to the presence of the occluder itself. Implantation of the occluder itself, as shown in PFO patients, did not alter ventricular motion pattern, as assessed by MR tagging.

